# Evaluation of factors influencing the guide to read biomedical English literature course for Chinese new medical postgraduates—a multiple regression analysis

**DOI:** 10.1186/s12909-019-1731-7

**Published:** 2019-08-01

**Authors:** Zhipeng Xu, Jingfan Qiu, Bingya Yang, Peng Huang, Lei Cai, Lin Chen, Min Hou, Minjun Ji, Guanling Wu

**Affiliations:** 1 Department of Pathogen Biology, Nanjing Medical University, Nanjing, Jiangsu 211166 People’s Republic of China; 2 School of Public Health, Nanjing Medical University, Nanjing, 211166 Jiangsu China; 3 Graduate school, Nanjing Medical University, Nanjing, 211166 Jiangsu China; 4 Teaching affairs office, Nanjing Medical University, Nanjing, 211166 Jiangsu China

**Keywords:** Educational measurement, Reading literature, Postgraduates, Medical

## Abstract

**Background:**

There is a dearth of published literature that demonstrates the impact of a Guide to Reading Biomedical English Literature course on new Chinese medical postgraduates. Keeping this gap in mind, the objectives of this study were to assess the factors associated with course effectiveness using the teacher, postgraduate and organizational factors.

**Methods:**

This study was conducted at Nanjing Medical University from December 2014 to December 2015. The participants were 440 new graduate students from different medical specialties. At baseline, each student was assessed for teacher factors, individual factors and organizational factors using a self-administered structured scored anonymous questionnaire. After that, Pearson chi-square analysis was conducted to evaluate the factors that impact teacher factors (knowledge level, teaching style, individualized teaching, logical teaching, heuristic teaching, literature difficulty, bilingual teaching), individual factors (gender, attitude toward studying, previewing literature, English literacy level) and course management (such as teaching objectives and assessment system) on this course. Furthermore, multiple logistic regression analyses were performed to determine the impact of the above factors on our outcome variables (knowledge level, teaching style, individualized teaching, heuristic teaching, study attitude, previewing literature, management).

**Results:**

Nearly all of the participants (420 of 440, 95.5%) thought this course was helpful for learning to read scientific literature and understanding scientific research design. Multivariate logistic regression analyses showed that the participants perception of the course as effective was associated with teachers’ high knowledge level (Adjusted Odds Ratio, AOR = 49.673; 95% confidence interval, 95% CI = 4.28, 575.90). In addition, heuristic teaching was found to be significantly associated with a positive teaching effect of teaching (AOR = 12.76; 95% CI = 1.78, 91.64). Furthermore, the participants perception of the course as effective was associated with positive attitude toward studying (AOR = 25.004; 95% CI = 2.51, 249.09). Previewing literature was also associated with course effectiveness (AOR = 0.02; 95% CI = 0.04, 0.11).

**Conclusions:**

This study indicated that the course effectiveness of the Guide for Reading Biomedical English Literature was associated with i) teachers’ knowledge, ii) heuristic teaching, iii) students’ positive attitude, and iv) students’ previewing literature.

**Electronic supplementary material:**

The online version of this article (10.1186/s12909-019-1731-7) contains supplementary material, which is available to authorized users.

## Background

Higher medical education in China has developed quickly, not only providing more clinical doctors and medical researchers for the country but also posing a significant challenge for postgraduates wishing to devote themselves to society [1]. Medical postgraduates must develop their abilities and professional skills, one of the most fundamental of which is literature reading competence at the university level.

As one scholar said, “If an organization does not learn faster than the rate of change in their environment, they die” [2], and the same applies to medical graduate students. Reading the scientific literature is not only an important part of medical research but also an essential part of postgraduate study. Studies have noted that students who have grown up in non-English speaking countries are always limited by language and professional knowledge in reading English literature, including difficulties in understanding and memory [3, 4]. This difficulty has become a major obstacle for postgraduates’ acquisition of relevant scientific information through reading. Keeping this in mind, Prof. Guanling Wu (Nanjing Medical University) established a course called “Guide to Reading Biomedical English Literature” in 2004. This course is designed to instruct each new postgraduate student in mastering how to read scientific literature and obtain effective scientific information from their reading.

The course, which is open to new postgraduates who are newly admitted to the university, is now a popular elective course for postgraduates at Nanjing Medical University. The lectures help graduate students master strategies, methods, and techniques for reading biomedical papers through lectures, examples and class discussions, which meet the requirements of conducting research projects. Since the beginning of the course in 2004, it has been welcomed by students because of its practicality and its novel and unique teaching methods. Students’ enthusiasm for this elective is high: the annual student elective rate (electives enrollment accounts for all new medical graduates) is approximately 60–80%, and each class has high attendance (good compliance). Through taking this course, a large number of new postgraduates can shift from passive acceptance of popular education in English to active learning of professional knowledge as soon as possible, thereby accelerating the process of reaching the forefront of international disciplines.

### Aim

We aimed to evaluate the factors that affect the effectiveness of implementing this course with medical postgraduates in China: 1) to estimate the association of teaching effectiveness with teacher, postgraduate and organizational factors; 2) to investigate the influencing factors on effective teaching outcomes of the course; and 3) to identify problems in the course and adjust the teaching plan in the future.

## Methods

### Participants

A cross-sectional survey was conducted in Nanjing Medical University, Jiangning District, Nanjing, in December 2014, with postgraduates who took the course “Guide to Reading Biomedical English Literature”. A total of 440 questionnaires were collected from 589 graduates for the analyses, yielding a response rate of 73.57%. All of the participants were first-year postgraduates, having completed five years of undergraduate study. It will take these students three years to get their master’s degree as postgraduates. The specialties of postgraduate students in our study included basic medicine, pharmacy, public health, nursing, social medicine, stomatology, and clinical medicine.

### Course schedule

This course (2 credits) has been a popular elective for new graduate students since 2004. It covers seven items: “strategies and techniques for reading English literature, document review of project planning and implementation stages, interpretation of dissertations, experience in developing writing skills from literature reading, review reading, critical reading, and application of literature in the writing of postgraduates’ research plans” (see Table 1); each item is taught by its own professor. Four hours are devoted to each item in this course (for a total of 28 h). Before this course, the course leader convened all teachers at a collective preparation and training session to develop teaching plans to exclude variation in teacher factors. Five speakers in this course used the Exemplary Teaching Method, which means teaching by example literature. All of the seven professors teaching in this course have more than 10 years of teaching experience and more than 1 year of overseas (American) study experience. This course’s assessment requires each student to submit answers to questions about the selected literature (see Additional file 1). Teachers scored these answers.

**Table 1:** Course Schedule of Biomedical English Literature Guide

Speakers	Contents	Teaching methods	Language
Speaker 1	Strategies and techniques for reading English literature	Lecture	Chinese
Speaker 2	Document review of project planning and implementation stage	Exemplary Teaching (Model Article 1)	Chinese-English
Speaker 3	Interpretation of the dissertation	Exemplary Teaching (Model Article 2)	Chinese-English
Speaker 4	Experience writing skills from literature reading	Exemplary Teaching (Model Article 3)	Chinese-English
Speaker 5	Review reading	Exemplary Teaching (Model Article 4)	Chinese-English
Speaker 6	Critical reading	Exemplary Teaching (Model Article 5)	Chinese-English
Speaker 7	Application of literature in the writing of postgraduates’ research plan	Lecture	Chinese

### Questionnaire

A questionnaire was designed to investigate the factors affecting the course effectiveness. Since this is the first study to determine the factors associated with the effectiveness of this course guiding Chinese new medical postgraduates in reading biomedical English literature (see Additional file 2), the study used a self-designed paper-based Chinese questionnaire that comprised 12 (8 + 3 + 1) questions (factors) regarding teacher factors (knowledge level, teaching style, individualized teaching, logical teaching, heuristic teaching, literature difficulty, bilingual teaching), student factors (gender, attitude toward studying, times of preview literature including translation of professional vocabulary and understanding of experimental methods, English literacy level), and course management (such as teaching objectives, organization, coordination, assessment system).

At the final class of this course, new postgraduates were asked to submit a questionnaire, which was completed anonymously in order not affect or bias respondents’ answers.

Demographic data were collected on gender, age, medical specialty. Further items asked participants to answer questions using clear, guiding language. The main research content includes four aspects: teacher’ factors, postgraduate’ factors, organizational factors, and the effect of teaching. All participants completed the questionnaire in the class. These questions were scored on a scale of 1 (High), 2 (Medium), or 3 (Poor); a numerical score; or Yes/positive (No/negative). The data was double-blind entered to ensure the accuracy of the questionnaire data.

### Statistical analysis

Data were entered into EpiData 3.0 and analyzed with SPSS 19.0 (SPSS Inc., Chicago, IL, USA). First, we listed the frequencies of basic characteristics of the graduates. Second, χ2 analyses were performed to assess the association of teaching effectiveness with teacher factors, postgraduate factors, and organizational factors. Third, to control for the confounding effects of our study variables, multivariate logistic regression analyses were conducted. In our logistic regression analyses, the variable was teaching effectiveness (yes/no), and the independent variables included teacher factors (knowledge level, teaching style, individualized teaching, logical teaching, heuristic teaching, teaching language), graduate student factors (times to preview literature, attitude toward studying, English literacy level), and the organizational factors (management). *P* < 0.05 was considered statistically significant in our study.

## Results

### Demographic data

The demographics of the study participants are shown in Table 2. The mean age of participants was 25.5 years old. Of the 440 participants, 181 (41.8%) students were male, and 256 (58.2%) were female. Fifty-six (12.7%) were aged below 25 years, 350 (79.5%) were aged from 25 to 26 years, and 34 (7.7%) were aged above 25 years.

**Table 2:** Basic characteristics of the graduates (*n* = 440)

Characteristics	n	%
Gender		
Male	184	41.8
Female	256	58.2
Age, years		
< 25	56	12.7
25~26	350	79.5
> 26	34	7.7
Type of medical specialty		
Clinical medicine	239	54.3
Basic medicine	113	25.7
Public health	33	7.5
Social medicine	18	4.1
Pharmacy	17	3.9
Stomatology	16	3.6
Nursing	4	0.9

The medical specialty distributions are as follows: 113 (25.7%) students are majoring in basic medicine, 17 (3.9%) students are majoring in pharmacy, 33 (7.5%) students are majoring in public health, 4 (0.9%) students are majoring in nursing, 18 (4.1%) students are majoring in social medicine, 16 (3.6%) students are majoring in stomatology, and 239 (54.3%) students are majoring in clinical medicine.

### Evaluation of the effectiveness of the course

Table 3 shows the effectiveness of the course after the new graduates took the course. Nearly all (420 of 440, or 95.5%) participants thought this course improved their reading ability and skills in reading and interpreting English medical literature, while 20 participants thought this course had no effect.

**Table 3:** Evaluation of the effectiveness of the course by graduates (*n* = 440)

	Number	Percent
Yes	420	95.5
No	20	4.5
Total	420	100

Table 4 shows the results of χ2 analyses examining the association of teaching effects with related factor. The results showed that several variables (logical teaching, difficulty of selected papers, bilingual teaching, gender, student’s English literacy level) had no significant association with teaching effect (all *p* > 0.05). However, other variables (knowledge level, teaching style, individualized teaching, heuristic teaching, attitude toward studying, previewing time, management) appeared to be associated with teaching effect (all *p* < 0.05).

**Table 4:** Association of effectiveness of this course with teachers, graduate students and organizational factors (*n* = 440)

Measures	Teaching effectiveness		χ2	*P*
	Yes (n, %)	No (n, %)		
Teachers factors				
Knowledge level				
High	390(92.9)	9(45.0)	46.236	0.000
Medium	30(7.1)	11(55.0)		
Teaching style				
High	297(70.7)	8(40.0)	12.419	0.002
Medium	116(27.6)	10(50.0)		
Poor	7(1.7)	2(10.0)		
Individualized teaching				
High	248(59.0)	6(30.0)	17.644	0.000
Medium	159(37.9)	10(50.0)		
Poor	13(3.1)	4(20.0)		
Logical teaching				
High	333(79.5)	14(70.0)	2.422	0.298
Medium	83(19.8)	5(25.0)		
Poor	3(0.7)	1(5.0)		
Heuristic teaching				
High	279(66.4)	3(15.0)	43.515	0.000
Medium	128(30.5)	11(55.0)		
Poor	13(3.1)	6(30.0)		
Literature difficulty				
High	84(20.0)	6(25.0)	1.256	0.534
Medium	242(57.6)	10(45.0)		
Poor	94(22.4)	4(30.0)		
Bilingual teaching				
Chinese	80(19.3)	3(15.0)	0.231	0.631
Chinese-English	334(80.7)	17(85.0)		
Student factors				
Gender				
Male	179(97.3)	241(94.1)	2.436	0.119
Female	5(2.1)	15(5.9)		
Attitude toward Studying				
Positive	411(97.4)	11(55.0)	78.781	0.000
Negative	9(2.1)	9(45.0)		
Times of preview				
< 1 time	3(0.7)	14(70.0)	86.431	0.000
1 time	94(22.4)	3(15.0)		
2~3 times	301(71.7)	3 (15.0)		
> 3 times	20(4.8)	0(0)		
English level				
CET 4	80(19.0)	4(20.0)	0.011	0.916
CET 6	340(81.0)	16(80.0)		
Organizational factors				
Management				
High	264(63.0)	7(35.0)	6.608	0.037
Medium	141(33.7)	11(55.0)		
Poor	14(3.3)	2(10.0)		

Note: all of the results were used continuity correction of Chi-Square Tests

After controlling for the covariates (age, education, area, safety knowledge and behavioral risk score), the participants’ perception of the course as effective was associated with teachers’ high knowledge level (AOR = 49.673; 95% CI = 4.28, 575.90). In addition, heuristic teaching, which means guiding graduate students to ask questions or asking graduate students to express their opinions, was found to be significantly associated with a positive teaching effect (AOR = 12.76; 95% CI = 1.78, 91.64). The participants’ perception of the course as effective was associated with positive attitude toward studying (AOR = 25.004; 95% CI = 2.51, 249.09). Literature previewing was also positively associated with course effectiveness in our study (AOR = 0.02; 95% CI = 0.04, 0.11) (Tables 5).

**Table 5:** Factors associated with course availability by multivariate logistic regression analysis (*n* = 440)

Variables	Sig.	Exp(B)	95% C.I	
			Lower	Upper
Knowledge level ^a^	.002	49.673	4.284	575.896
Teaching style ^a^	.822	1.203	.241	5.993
Individualized ^a^	.371	1.962	.448	8.587
Heuristic teaching ^a^	.011	12.760	1.777	91.636
Times of preview ^a^	.000	.020	.004	.108
Study attitude ^a^	.006	25.004	2.510	249.090
Management ^a^	.953	.958	.225	4.069

Note: ^a^ Models were adjusted by knowledge level, teaching style, individualized teaching, heuristic teaching, study attitude, times of preview, and management

The reference category of knowledge level, teaching style, individualized teaching, heuristic teaching, study attitude, times of preview, and management were high level of knowledge, good teaching style, high individualized teaching, high heuristic teaching, positive study attitude, less times of preview, and highly management

In addition, the graduate students made some suggestions for improvement, including the following: 62% of the students believe that it is necessary to reduce the difficulty of the selected papers and choose some articles that are short and easy to understand; more than 75% of the students recommended including the teachers’ experience in scientific research; and 65% of the students suggested increasing English writing skills training.

## Discussion

The education of medical postgraduates shoulders the dual responsibility of cultivating high-quality medical talents and developing medicine related science and technology, which is closely related to the sustainable development of the medical field in China. Training innovative talents, as one of the major aims for universities in China, requires the reform of traditional teaching methods [5]. However, in the field of medical school, little research is oriented to courses that guide new postgraduates in reading scientific literature. For that reason, this paper is the first attempt to study the effectiveness of such a course in postgraduate-centered higher education, especially the relationship between course effectiveness and the teacher, postgraduate and organizational factors, such as teachers’ knowledge or heuristic teaching and students’ attitudes or literature previewing.

The new course has been welcomed and is well-attended by new graduate students; for example, the proportion of postgraduate students participating in the survey of this course accounted for 73.57% (440/589) of all postgraduate students in 2014. After taking this course, more than 95% of the graduate students believe that they have mastered literature screening and have learned reading skills and strategies (intensive or extensive reading, critical reading, problem-based reading and thinking, collation and classification of documents, etc.), which play important roles in aiding the design and implementation of their research topics. Marušić et al.’s study [6] was mainly focused on study skills, such as access to medical literature and bibliographic databases, and taught young academic physicians to write a scientific article; while our work was mainly study the factors associated with course effectiveness from teachers, postgraduates and organizational factors.

The students’ ability and skill in reading medical literature will be greatly improved after teachers are professionally trained. This new course for guiding students in reading biomedical literature is challenging for teachers, particularly those who teach using traditional methods. How teachers control a course and how they act in the classroom are key influences on learning outcomes. Indeed, our study showed that teachers’ knowledge level and heuristic teaching were associated with enhancing the effectiveness of this course. The breadth of a teacher’s knowledge of basic and clinical is one of the most important influences on the work performed in classrooms and, ultimately, on what medical students learn [7]. We speculated that teachers who have rich knowledge about medicine at the organ, cellular, and molecular levels, as well as of clinical cases, may improve the effectiveness of this course.

Another requirement associated with effectiveness of this course was to encourage heuristic teaching. The old Chinese proverb states, “Give a man a fish and you feed him for a day. Teach him how to fish and you feed him for a lifetime”. Heuristic teaching is not a specific teaching method, but an idea that guides teaching practice. This approach emphasizes the development of students’ intelligence and, promotes students’ internal learning motivation. In addition, heuristic teaching is a teacher-led and student-centered interaction, encouraging students to actively complete the teaching and learning process [8, 9]. In our course, teachers told the students in advance about the English papers selected for the next class in advance and provided several key questions to help them to prepare. In class, several representative graduate students were asked to answer the key questions, and the other graduates supplemented with their own, which ensured that each student got involved. Heuristic teaching not only improved students’ ability to find and solve problems but also created an active atmosphere in Guide to Reading Biomedical English Literature classroom, which inspired graduate students’ interest in learning and strengthened their mastery of document reading.

In addition, students’ understanding of the progress of a course greatly influences their learning outcomes. The Doctrine of the Mean by Confucius states, “Success depends upon previous preparation, and without such preparation there is sure to be failure” [10]. Previewing the literature is an important part of this course, which included translation of professional vocabulary, understanding of document methods and techniques, understanding of experimental methods and principles, reading of reference materials, and summarization. This approach is different from flipped classrooms, which mainly foster student ownership of learning through the completion of preparatory work and being more interactive during actual class time. In addition, flipped classrooms allow students to learn at their own pace and give them the flexibility to use electronic resources [11]. In our study, we found that previewing literature is effective. However, we think it appropriate to consider flipping the classroom in the implementation of this course in future. Most medical graduate students’ perceptions of research are largely positive, as they perceive that their rigorous academic experiences contributed to their career progression, helped them to identify their career paths [12, 13], increased their confidence regarding their professional position [14], and provided an opportunity to integrate and apply their findings in their practice [15, 16]. Consistent with these finding, our study showed that a positive attitude toward studying was associated with course effectiveness. Indeed, students’ positive attitudes toward research increased their interest in applying principles they learned to the practice of medicine [17]. Although Chinese medical postgraduates encounter a substantial language obstacle in their academic pursuits [18], our study showed that the English literacy level, which is known as the “College English Teaching (CET)” level in China [19], was note shown to not influence the effectiveness of the course, likely because the college students’ English literacy level has significantly improved in China in recent years [20].

Although our study found that the course management factors, such as teaching objectives, organization, coordination, and assessment system, have no impact on the course effectiveness, we believe that good course management practices and course reforms are needed to improve course efficiency in future. Indeed, in order to enhance the effectiveness and influence of this course, we have already started to organize and implement Small Private Online Courses (SPOCs) in multiple medical schools in China.

This study has some limitations. Since this course is an optional elective rather than a compulsory course, participants from a self-selected group are interested in learning English literature. Thus, the evaluations and recommendations in the questionnaires received may not represent all student groups in our university.

## Conclusions

New postgraduates enrolled in medical school felt they effectively understood the scientific and scientific research design after taking the Guide to Reading Biomedical English Literature course. The students thought the course performed well when the teacher was knowledgeable and when teaching was heuristic. However, several factors evinced little differences, including logical teaching, difficulty of papers selected, bilingual teaching, teaching style, individualized teaching, gender, students’ English literacy level, and course management. The findings suggested that integration of medical knowledge into heuristic teaching could improve the effectiveness of the course. It is also particularly important to develop graduates’ self-learning ability in medical school. Based on our study, we think it appropriate to consider possible interventions for the course in future, especially a focus on teachers’ knowledge and heuristic teaching methods, students’ attitude and motivation, and the introduction of flipped classrooms.

## Additional files

Additional file 1: Final Examination. (DOCX 23 kb)

Additional file 2: Questionnaire for the Literature Guide. (DOCX 25 kb)

## Data Availability

The datasets during and/or analyzed during the current study are available from the corresponding author on reasonable request.

## Abbreviations

95 % CI: 95% confidence interval
AOR: Adjusted Odds Ratio
CET: College English Teaching
SPOC: Small Private Online Course

## References

[1] Zheng S, Shi S (2016). Medical education and medical professionalism in China. Lancet..

[2] Ifeoma EA, Rebecca EF, Ezekiel OO (2015). A cross-sectional study of the knowledge and attitude of medical laboratory personnel regarding continuing professional development. Niger Med J.

[3] Hubbard KK, Blyler D (2016). Improving academic performance and working memory in health science graduate students using progressive muscle relaxation training. Am J Occup Ther.

[4] Viana Ricardo Borges, Campos Mário Hebling, Santos Douglas de Assis Teles, Xavier Isabela Cristina Maioni, Vancini Rodrigo Luiz, Andrade Marília Santos, de Lira Claudio Andre Barbosa (2018). Improving Academic Performance of Sport and Exercise Science Undergraduate Students in Gross Anatomy Using a Near-Peer Teaching Program. Anatomical Sciences Education.

[5] The L (2017). Medical education reform in China. Lancet..

[6] Marusic A, Marusic M (2003). Teaching students how to read and write science: a mandatory course on scientific research and communication in medicine. Acad Med.

[7] Koens F, Custers EJ, ten Cate OT (2006). Clinical and basic science teachers' opinions about the required depth of biomedical knowledge for medical students. Med Teach.

[8] Alvarez Manilla J (1974). Methodology for the teaching of medicine in the future. Use of models, heuristic methods and self-teaching systems. Educacion medica y salud.

[9] Dolcini MM, Canin L, Gandelman A, Skolnik H (2004). Theoretical domains: a heuristic for teaching behavioral theory in HIV/STD prevention courses. Health Promot Pract.

[10] Yuan B, Shi D (2002). Influence of Confucian’s doctrine of the mean on formulae of traditional Chinese medicine. Zhonghua Yi Shi Za Zhi.

[11] Kowalski K, Horner MD (2015). Preparing educators to implement flipped classrooms as a teaching strategy. J Contin Educ Nurs.

[12] Gotterer GS, O'Day D, Miller BM (2010). The emphasis program: a scholarly concentrations program at Vanderbilt University School of Medicine. Acad Med.

[13] Chang Y, Ramnanan CJ (2015). A review of literature on medical students and scholarly research: experiences, attitudes, and outcomes. Acad Med.

[14] McPherson JR, Mitchell MM (1984). Experience with providing research opportunities for medical students. J Med Educ.

[15] Burgoyne LN, O'Flynn S, Boylan GB (2010). Undergraduate medical research: the student perspective. Med Educ Online.

[16] Jacobs CD, Cross PC (1995). The value of medical student research: the experience at Stanford University School of Medicine. Med Educ.

[17] Zier K, Friedman E, Smith L (2006). Supportive programs increase medical students’ research interest and productivity. J Investig Med.

[18] Tan X, Chen M, Chen X, Liao R, Sun J (2015). A shift of focus is required in English courses for Chinese medical postgraduates. Med Teach.

[19] Guo Q, Sun W (2014). Economic returns to English proficiency for college graduates in mainland China. China Econ Rev.

[20] Zhang L, Zhang W, Wu C (2017). Undergraduate medical academic performance is improved by scientific training. Biochem Mol Biol Educ.

