# Supplementary material for: Evaluation of factors influencing the guide to read biomedical English literature course for Chinese new medical postgraduates—a multiple regression analysis
Source: BMC Med Educ. 2019 Aug 1;19:295. doi: 10.1186/s12909-019-1731-7 (PMC6676574; doi:10.1186/s12909-019-1731-7)
Supplement: Supplementary file 1 — Final Examination. (DOCX 23 kb) [file 12909_2019_1731_MOESM1_ESM.docx]

**Additional file 1.** **Final Examination**

Name: ________ Student ID: ________ Score: ________

Please read the assessment document "________" and complete the following questions.

1.Paper title:

2.Key words:

3.Research aim:

4.Main findings:

5.Experimental control design:

6.Technical roadmap in the paper:

7.Conclusion:

8.Strengths and shortcomings (if any):

9.Combine your major and talk about what inspired you from this document:
