# Supplementary material for: Evaluation of factors influencing the guide to read biomedical English literature course for Chinese new medical postgraduates—a multiple regression analysis
Source: BMC Med Educ. 2019 Aug 1;19:295. doi: 10.1186/s12909-019-1731-7 (PMC6676574; doi:10.1186/s12909-019-1731-7)
Supplement: Supplementary file 2 — Questionnaire for the Literature Guide. (DOCX 25 kb) [file 12909_2019_1731_MOESM2_ESM.docx]

**Additional file 2. Questionnaire for the Literature Guide**

1、How old are you？________

（1）<25 （2）25~26 （3）>26

2、What is your medical specialty________

（1）Clinical medicine （2）Basic medicine （3）Public health

（4）Social medicine （5）Pharmacy （6）Stomatology

（7）Nursing

3、Do you think that such a course can improve your reading level and skills in medical English literature? __________

（1）Yes （2）No

4、What do you think of knowledge level of the teachers in this course? __________

（1）High （2）Medium

5、What do you think of teaching style, such as humorous or freedom of thought in the course? __________

（1）High （2）Medium （3）Poor

6、What do you think of individualized teaching in the course? __________

（1）High （2）Medium （3）Poor

7、What do you think of logical teaching in the course? __________

（1）High （2）Medium （3）Poor

8、What do you think of heuristic teaching (guide graduates to ask questions or induce graduates to express their opinions) in the course?

（1）High （2）Medium （3）Poor

9、How do you think the difficulty of selecting the literature? __________

（1）High （2）Medium （3）Poor

10、What language is the teacher of the course suitable for? __________

（1）Chinese （2）Chinese-English

11、What is your gender__________

（1）Male （2）Female

12、What do you think of your attitude towards this course? __________

（1）Positive （2）Negative

13、How many times of preview the medical literature before class? __________

（1）<1 time （2）1 time （3）2~3 times （4）>3 times

14、What is your English level? __________

（1）CET 4 （2）CET 6

15、How do you think about the level of management (teaching objectives, organization, coordination, assessment system, etc.) of this course? __________

（1）High （2）Medium （3）Poor

16、Do you have some suggestion for this course?
